# Yoga-Based Group Intervention for In-patients With Schizophrenia Spectrum Disorders—A Qualitative Approach

**DOI:** 10.3389/fpsyt.2021.715670

**Published:** 2021-08-13

**Authors:** Theresa Schulze, Eric Hahn, Inge Hahne, Niklas Bergmann, Lukas Marian Fuchs, Franziska Mähler, Marco Matthäus Zierhut, Thi Minh Tam Ta, Gerdina Hendrika Maria Pijnenborg, Kerem Böge

**Affiliations:** ^1^Department of Psychiatry and Psychotherapy, Campus Benjamin Franklin, Charité – Universitätsmedizin Berlin, A Corporate Member of Freie Universität Berlin, Humboldt-Universität zu Berlin, and Berlin Institute of Health, Berlin, Germany; ^2^Department Clinical Psychology and Experimental Psychology, Faculty of Behavioral and Social Sciences, University of Groningen, Groningen, Netherlands; ^3^Institute of Sociology, Freie Universität Berlin, Berlin, Germany

**Keywords:** yoga therapy, schizophrenia spectrum disorder, qualitative study, body-mind therapies, complementary therapies, mechanisms, mindfulness, embodiment

## Abstract

**Background:** Yoga may pose a promising complementary therapy in the multimodal treatment of in-patients with schizophrenia spectrum disorders (SSD). However, to date, no studies have qualitatively examined in-patients' with SSD experiences of Yoga as well as their perceptions of its limitations and benefits as a treatment component. This qualitative study aimed to explore for the first time the mechanisms and processes of Yoga-based Group Intervention (YoGI) for in-patients with SSD in Germany by asking for their subjective experiences. Findings could serve as a preliminary basis for developing an effective and evidence-based YoGI manual tailored to this patient group.

**Materials and Methods:** In total, 25 semi-structured interviews were conducted directly after YoGI, for which responses were either noted down by hand or audio-recorded. The interview guide was pilot-tested and consisted of 14 questions to explore the personal articulated experiences of participation in YoGI from in-patients with SSD. Positive, negative, depressive, and anxiety symptoms were assessed during a diagnostic interview and through questionnaires. The interview data was transcribed, coded by two independent researchers, and analysed using an inductive thematic approach. The research team collaboratively discussed emerging categories to reduce redundancy and form meaningful themes and subthemes.

**Results:** The analysis revealed seven main themes. YoGI was perceived as feasible and focusing on individual adaptation, captured by the theme *inclusivity*. Nevertheless, participants encountered *challenges*; thus, physical limitations need to be considered. While practising together, participants experienced *interconnectedness* and developed a *mindful stance* as they accepted their limitations and adapted exercises with self-compassion. Patients described that following the flow of the asanas required physical persistence, which ultimately led many participants to experience *confidence* and *relaxation*. YoGI affected *symptom representation* as heightened awareness led participants to notice impeding as well as improved symptoms.

**Conclusion:** YoGI showed various promising effects on in-patients with SSD. Future research should examine to what extent these effects can be sustained and how the mindful approach during YoGI can be transferred to areas outside the Yoga class. Furthermore, a randomised controlled trial could investigate the effectiveness of a manualised YoGI.

## Introduction

As the first line of treatment, patients with schizophrenia spectrum disorders (SSD) usually receive antipsychotic medication, which seems to be especially effective for the treatment of positive symptoms (PS) of schizophrenia such as hallucinations and delusions (1, 2). Despite adequate pharmacological treatment, however, a considerable proportion of patients does not show any symptom improvement (3, 4). While the latest German treatment guidelines recommend Cognitive-Behavioural-Therapy to be offered to all patients (5), it is only received by a few (6). Especially negative symptoms (NS), consisting of blunted affect, diminished speech, anhedonia, social withdrawal, and lack of motivation (7), as well as cognitive dysfunctions, remain challenging to the available treatment options. The persistence of NS and cognitive dysfunctions emphasises the need for researching and implementing complementary treatments for this patient group within a multidisciplinary strategy. Yoga as an adjunctive therapy has been growing in research and application in mental health care in general and just recently for patients with SSD specifically (8, 9).

Yoga, a philosophical doctrine that originated in ancient India and encompassed mental, physical and spiritual practises, has been increasingly adopted in the form of a secular mind-body therapy in the West (10–12). Studies show that Yoga Therapy (YT) constitutes a suitable add-on treatment for psychiatric disorders because it can be easily implemented, is cost-effective, and can be instructed by various professionals working in mental health (10). YT (13) is composed of breathing exercises (*pranayama*), postures (*asanas*), and relaxation (*shavasana*) and is usually delivered in a group setting.

From a linguistic perspective, the term Yoga is derived from the Sanskrit word *yuj*, meaning “*to yoke together*” or “*to unite*.” Concurrently, the linguistic development of the term schizophrenia moved toward a more integrative understanding of the disorder focusing on the failure on integration (14): Schizophrenia was renamed *integration disorder* in Japan (15) and *attunement disorder* in South Korea (16). Thus, Yoga, as a form of (re-)connexion may target disintegration and hence, seems intuitively applicable for a modern treatment approach for SSD. The symptomatology and underlying processes have been described as a disconnection syndrome at several levels of brain networks (17), and functionally as a disintegration of mental processes, affect but also interpersonal relationships (18). Further, YT might be especially suitable to be offered to in-patients with SSD because it has positive effects on clinical insight and medication adherence (19). Better medication adherence might prevent relapses, benefit the overall course of the illness, and reduce the chance of future hospitalisations. During hospitalisations, YT can reduce stress in acutely ill patients, which is of crucial importance at the beginning of their treatment (20, 21) when symptoms and difficulties tend to be exacerbated. Already after a single yoga session, people with SSD display decreased state anxiety, reduced psychological stress, as well as increased subjective well-being (22). Furthermore, studies report increased quality of life for up to 6 months after YT (23, 24). Moreover, studies on YT show improvements in cognition, including attention precision and speed in abstraction, attention (25), and memory (26). Also, facial-emotion recognition (27, 28) improved after YT, which is linked to increased socio-occupational functioning (27–29). In fact, recent research found that persons with schizophrenia who attended a YT over the course of 6 weeks significantly improved in social cognition performance compared to those in the waitlist control group (30).

The empirical evidence of YT on the general psychopathology of SSD seems to be more heterogeneous: A meta-analysis in 2018 reports that mindful exercises have more beneficial outcomes on psychiatric symptoms than non-mindful exercises, and Yoga specifically seemed to be a promising intervention for NS (31). Meta-analyses from the following year obtain similar results, concluding that mind-body therapies, including Yoga, can improve NS, yet effects are small (32) to moderate (33) and display high heterogeneity. But even small effects might be of clinical relevance because NS pose a great subjective burden to people with SSD who describe the experience of losing concentration and motivation, withdrawal, and numbness feelings as disabling and persistent (34). A more recent meta-analysis showed moderate positive effects of mind-body exercises, including Yoga, on PS, NS, as well as depression in patients with schizophrenia (35). People with SSD receiving YT in in-patient (29, 36) and outpatient care (27, 29) both experienced reductions in PS, NS as well as general psychopathology. According to the NICE treatment guidelines for psychosis and schizophrenia in adults (37), Yoga can, compared with aerobic physical activity, improve quality of life at short term follow up as supported by one study with high quality evidence (29). However, a majority of randomised controlled trials are of low quality and research on YT for patients with SSD is methodologically flawed by missing or inadequately reported outcomes and procedures (38, 39). According to a Cochrane Review, there is currently not sufficient high-quality evidence to neither support nor discourage the use of YT as an adjunctive treatment for schizophrenia (40), emphasising the need for further research.

Various schools of Yoga highlight different elements, yet despite this heterogeneity, YTs show no significant differences in their effects (41) and they might share common underlying factors inherent to Yoga or other mind-body therapies exerting these effects. One of these factors could be mindfulness (42). Yoga is also referred to as “mindfulness in motion” (43) because one moves with intentional awareness through various poses while being anchored in ones' breath. This embodied practise of mindfulness, unifying the mind and body through the breath, might lead to an experience of re-connexion on several levels (18), possibly affecting practitioners lives beyond the yoga mat. Therapies integrating mindfulness-based approaches can provide guidance in coping with symptoms rather than curing these (44), which is especially relevant in the treatment of patients with SSD with a chronic course. Dealing with symptoms in a mindful way includes acting with awareness of the present moment experience and having a non-judgmental and non-reactive attitude (45, 46). Present moment awareness is achieved by placing awareness on experiences related to body, feelings, mind, and phenomena (47). Yoga extends the role of the body as present moment awareness is inherently linked to entering different asanas, yet Yoga, and other mind-body therapies, have been rarely implemented in Western medical care until recently (12, 43).

Consequently, research on YT for patients with SSD is still limited in scope and due to the wide variety of Yoga styles, participants, as well as outcomes examined (32, 33, 38), it remains difficult to obtain conclusive evidence on the underlying mechanisms of YT. Particularly regarding this patient group, there is only one qualitative study of in-patients with psychotic disorders whose YT experience was characterised by relaxation, calm/reduced stress, improved energy/focus, and motivation to engage with life (48). These findings provide the first insights into working mechanisms of YT for patients with psychotic disorders, yet more qualitative research involving in-patients with SSD is needed to gain a deeper understanding of their experience of YT and its underlying factors.

Despite the therapeutic potential of Yoga for patients with SSD, the current body of research on Yoga as an add-on treatment for SSD lacks the perspective of this patient group. Primarily, the present study aims to close this gap by conducting semi-structured interviews to explore the subjective experiences of a newly developed **Yo**ga-based **G**roup **I**ntervention (**YoGI**) by in-patients with SSD. The close iterative process allows for gaining an in-depth understanding of the mechanisms and processes underlying YoGI and possibly provides novel impulses for further research and treatment adaptions. Furthermore, the participants' feedback is used to address current limitations concerning the applicability, practicality, and utility of this intervention. Thereby, this study is a first contributing step to an evidence base necessary for developing a YoGI manual specifically tailored to patients with SSD receiving treatment in a hospital setting.

## Materials and Methods

### Design

A qualitative approach was chosen to yield an in-depth understanding of in-patients with SSD experiences with YoGI. In addition, quantitative measures were obtained with the intention to provide a comprehensive clinical characteristics description allowing the assessment of exclusion criteria.

### Participants and Procedure

Participants were recruited from the in-patient ward for psychotic disorders of the Charité -Universitätsmedizin Berlin, Department of Psychiatry and Psychotherapy at Campus Benjamin Franklin. Patients in the age between 18 and 65 years with an ICD-10 F2x-spectrum-diagnosis, who received in-patient care at the ward for psychotic disorders and who provided informed consent were eligible to participate in the study. Patients who presented with severe psychotic symptoms, as indicated by any item ≥6 on the Positive Syndrome Subscale of the Positive and Negative Syndrome Scale (PANSS) (47), or who displayed a severe neurological disease assessed by a licenced psychiatrist were excluded from participation.

**Figure 1:**
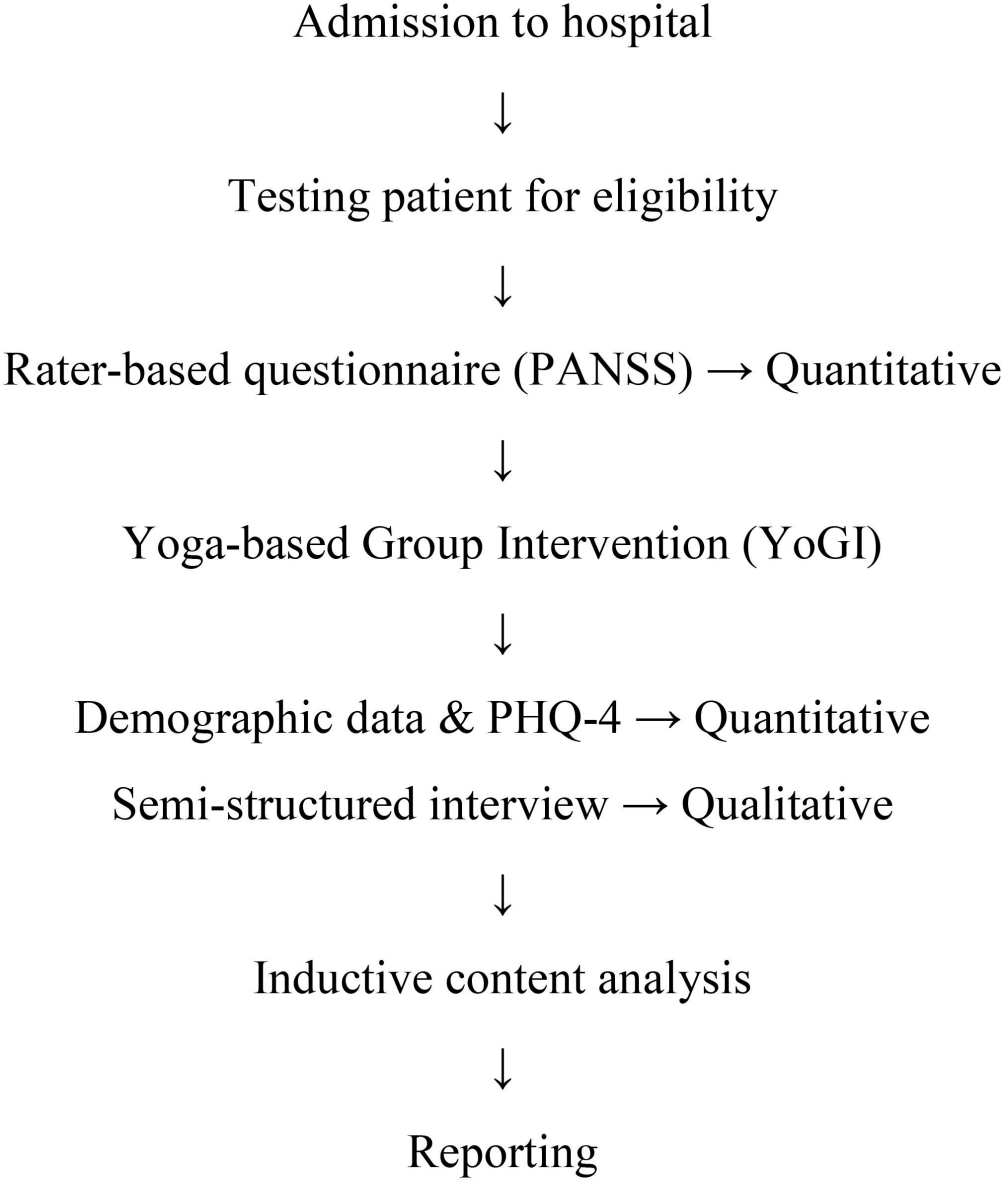
Study flow diagram.

At the ward, 29 patients who fulfilled the inclusion criteria were approached by the interviewer, who informed them about YoGI and the possibility of participating in a study afterwards. Four patients declined to try YoGI due to lack of motivation, disinterest in Yoga and/or meditation, as well as concerns about required levels of fitness and flexibility. Since patients needed to attend at least one session of YoGI, those who refused to engage in the intervention could not participate in the study either. However, all patients who took part in YoGI and were offered participation provided informed consent to participate in the data collection process taking place at two timepoints. First, the PANSS interview was administered within a week of attending YoGI. The PANSS interview was conducted by an experienced psychologist to determine the presence and severity of PS and NS as well as general psychopathology. Secondly, directly after attending YoGI, participants were invited to take part in a subsequent one-on-one interview session. In this session, demographic data such as age, sex, living situation, occupation, and medical information, including diagnosis, the onset of the disorder, length of stay at the hospital and psychiatric medication were assessed. Afterwards, the Patient Health Questionnaire (PHQ-4) (48) was administered to assess symptoms of depression and anxiety. Finally, the interview was conducted, transcribed, and analysed by means of inductive thematic analysis (see Figure 1). Respondents received no compensation for participating in the study. The study was approved by the ethical committee of the Charité Universitätsmedizin Berlin, EA4/169/20.

### Quantitative Measures

The Positive and Negative Syndrome Scale (PANSS) (47) is a semi-structured interview that assesses global psychopathology, PS, and NS. The standard version was used consisting of 16 items for global psychopathology (cronbach's alpha = 0.55), seven items for PS (cronbach's alpha = 0.62) and seven items for NS (cronbach's alpha = 0.92), all subscales display moderate to high internal consistency (49). The Patient Health Questionnaire (PHQ-4) (48) is a brief 4-item questionnaire to assess depressive and anxious symptoms and displays good internal consistency (cronbach's alpha = 0.85).

### Intervention

YoGI took place as part of an extensive interdisciplinary treatment programme for in-patients with SSD, including inter alia pharmacological treatment, individual- as well as group psychotherapy, occupational therapy, and exercise therapy. YoGI was offered on a weekly basis and patients were able to join the intervention at any time as sessions did not consecutively build upon another. Group sizes ranged from 3 to 10 participants and interviewees participated in different sessions over the course of 6 months. YoGI had a duration of 50 min and began with breathing exercises (*pranayama*) and checking in with one's own body. This was followed by a short round in which each participant briefly verbalised their current state of mind, body, or set an intention for class. Afterwards, the participants were guided through various *asanas* (exemplary YoGI protocol in Supplementary Materials) supported with mindful instructions that emphasised noticing any bodily sensations and explicitly instructed breathing in and out. YoGI ended in *shavasana*, yet instead of relaxing in silence, the final relaxation was guided by body scan instructions.

### Interviews

The qualitative data were collected by means of semi-structured interviews that aimed to explore the subjective experience of a YT. The initial list of questions was based upon a semi-structured interview guide investigating a Mindfulness-based group therapy for in-patients with SSD (50). The set of questions was refined in an iterative process with the patients during a pilot phase of five interviews which led to slight adaptations of the order and phrasing of questions. A team of researchers discussed the interview guide to ensure a comprehensive set of questions while reducing redundancy. Subsequently, a final list of 14 questions was established (see Table A1 in Appendix). The questions were phrased openly to allow participants to express what they considered most relevant. For example, participants were asked to 1. “*Describe what first comes to mind when thinking of YoGI*” and 5. “*How and what did you perceive during the exercises*.” Other questions invited participants to share 4. feelings and 6. potential challenges and report on 14. their current symptoms. Furthermore, specific questions such as 8. “*What did you manage well and what did you like?*” and 12. “*What did you miss? What could have been done better or differently?*” aimed at gathering opinions on what elements of YoGI were considered as either valuable or in need of adjustment.

### Data Collection and Storage

In total, the present study collected full data from 25 interviewees to represent a comprehensive account of the subjective experience of YoGI of in-patients with SSD. A sample size of *N* = 25, including five interviews conducted in the pilot phase, was chosen because code saturation and meaning saturation are reached after nine and 16–24 interviews, respectively (53), and guidelines consider sample sizes of around 25 as adequate number (54). In this sample the five interviews conducted in the pilot phase were included, which we deemed appropriate since only minor changes have been applied to the interview guide. The data collection process was started on the 26th of May 2020 and finished on the 14th of October 2020. Interviews were conducted after participating in at least one but no more than three sessions of YoGI. On average, the semi-structured interviews lasted 42 min, ranging from 25 to 70 min. Before the one-on-one interviews, participants were explicitly asked for consent to either have their answers noted down by hand or audio-recorded by the interviewer. Six interviewees opted for the first option and 19 for the latter. Moreover, interviewees chose a place to conduct the interview, 17 respondents wanted to stay in the room where YoGI took place, and eight decided to sit down outside in the park of the hospital to be interviewed. The interviews were conducted by TS, a female psychologist at the ward for psychotic disorders who has practised Yoga for several years and co-instructed YoGI together with IH, a licenced psychologist. The interview process and the instruction of YoGI were supervised by a team of psychologists and a licenced psychotherapist on a regular basis. Participants might have been in contact with the interviewer during other group therapies or studies offered at the ward before attending YoGI. The interviewer informed participants about the nature of the intervention and its embedment in the research for her thesis project.

Data was securely stored in locked cabinets, and electronic data was password protected. The pseudonymisation of participants' data was recorded on a separate, password-protected list. All data were accessible only by members of the research team. After completion of data collection, the decoding list, as well as any handwritten data, was deleted. Hence, only pseudonymous data will be available, which prevents any re-identification. The data will be stored for a maximum period of 10 years at the Charité–Universitätsmedizin Berlin, after which it will be deleted.

### Data Analysis

Illness severity scales (PANSS, PHQ-4) and sociodemographic data were analysed using Microsoft Excel 365 for Windows 10. The qualitative data from the semi-structured interviews were analysed following an inductive thematic approach. The coding was done in MAXQDA 2020 for Windows 10. The goal of the inductive thematic analysis was to organise identified categories into a model to summarise the raw data and highlight key themes (55, 56). The inductive thematic analysis followed the six phases outlined by Braun and Clarke (57). First, all interviews were transcribed using the same formatting. Afterwards, the transcripts were read and re-read by TS and FM who were involved to various degrees in the research process. Once the two readers were familiar with the text and reached a good understanding of its themes, they generated initial codes in a ground-up approach while reading (*in vivo* coding) across the entire data set in the second phase. In the third phase, codes were collated into potential themes. In phase four, these themes were reviewed as it was checked whether themes work in relation to the coded segments and the whole data set. Themes were clearly defined and named in the fifth phase. In the final phase, representative quotes of the interviewees were selected, translated from German to English, and the report was produced.

## Results

### Participant Characteristics

In the present study, 25 participants (12 identified with the female, 13 with the male, and no one identified with the diverse gender), with a mean age of 41.9 years (SD = 13.98), from the inpatient-ward for psychotic disorders were included. Of these, 17 participants were diagnosed with schizophrenia (F20), five with schizoaffective disorder (F25), two with delusional disorder (F22), and one with acute polymorphic psychotic disorder (F23), all according to ICD-10 criteria. Except for one participant, all received psychotropic medication. On average, participants showed moderate PS and NS and severe depressive and anxious symptoms. A detailed summary of sample characteristics is presented in Table 1.

**Table 1:** Sample characteristics (*n* = 25).

	**M**	**SD**	**Range**
Age (years)	41.9	13.98	20–64
Length of current hospital stay (days)	63.44	33.63	13–146
PHQ-4 (*n* = 24)	10.29	1.14	4–16
PANSS, Positive Syndrome Scale (*n* = 24)	16.04	5.14	8–27
PANSS, Negative Syndrome Scale (*n* = 24)	17.54	7.02	7–32
PANSS, General Psychopathology Scale (*n* = 24)	32.95	8.93	19–52

*M, mean; SD, standard deviation; PANSS, Positive and Negative Syndrome Scale (51); PHQ-4, Patient Health Questionnaire (52)*.

### Qualitative Results

During the interviews, respondents foremost reported their experiences of the previous YoGI session, and many gave detailed feedback on poses they particularly liked or that they struggled with. Most interviewees had neutral to positive attitudes towards Yoga before participating and except one participant, all reported to have felt relaxed at some point and to have benefitted from the intervention. Prior Yoga experiences ranged from non-existent to multiple years of following a regular individual practise; however, none of the participants had been practising Yoga regularly in the near past or were offered to practise Yoga in a therapeutic context before.

In the inductive thematic analysis, seven main themes emerged from the semi-structured interviews that describe participants' subjective experience of YoGI. Overall, YoGI was characterised by **inclusivity**, as the intervention was perceived as feasible and encouraged individual adaptation at all times. Yet, participants also encountered **challenges** during the practise related to physical limitations or the ability to engage with the intervention. YoGI affected **symptom representation** as heightened awareness led participants to notice impeding as well as improved symptoms. Through practising Yoga in a group, participants experienced a sense of **interconnectedness**. However, YoGI did not only strengthen participants' awareness for connection among each other but also to themselves. They developed a **mindful stance** as they accepted their limitations and adapted exercises with self-compassion. Through following the flow of asanas, participants were guided into trying new postures that resulted in building **confidence** and synchronising movement and breath helped many to enter a **state of relaxation**. A summary of themes, subthemes, and the corresponding codes can be found in Table 2.

**Table 2:** Themes, subthemes, and codes.

**Themes**	**Subthemes**	**Codes**
**Inclusivity**	*Feasibility*	Mix of exercises Level of difficulty Length of session Easy participation Different levels of prior experience Calm and safe setting
	*Adaptation*	Adapting exercises Instruction to individually adapt practise Adaptation chair Yoga Tailored instructions Experimenting with one's body Breathing in own rhythm
**Confidence**	*Motivation*	Motivation to practise Motivation to continue Yoga Motivation for other activities
	*Self-efficacy*	Self-knowledge Insight interplay body, mind, breath Empowered Self-confidence
	*Feeling achievement*	Feeling achievement Proud Content Flow
	*Sense of competence*	Physical perseverance Physical activity helps focus Sufficient concentration Learning and progress Trying new things and challenging oneself
**State of relaxation**		Calm mind Relaxed body Feeling relaxed Interplay tension relaxation Challenging elements Relieving tension Relaxing exercises Relaxed music Closing eyes
**Mindful stance**	*Awareness*	Body awareness Loss of ability Breath Present moment
	*Acceptance*	Acceptance of condition Accepting own limitations
	*Self-compassion*	Self-care No pressure to achieve Listening to oneself
	*State of oneness*	
**Interconnectedness**	*Dialogue with instructor*	Appreciating instructions Watching instructor Listening to instructions Empathic attitude
	*Practising and learning together*	Practising together Awareness of others Comparing oneself Imitating others Group helps with motivation
	*Feeling connected*	Empathy
		Connecting to and Appreciating Others Acknowledging Others Experience
**Challenges**	*Physical limitations*	Pain Overweight Dizziness
	*Engaging in practise*	Breathing rhythm irritating Understanding exercises Starting practise Interruptions by others
**Symptom representation**	*Ease of symptoms*	Well-being Lifted mood Improved pain Distraction from symptoms Distance to problems and worries Less strain through voices Fewer thoughts Bliss afterwards Improved interactions afterwards
	*Symptoms impeding practise*	Difficulty concentrating Experiencing hallucinations Mistrust Demotivation

#### Inclusivity

The theme **inclusivity** depicts that YoGI is an inclusive practise as it displays *feasibility* for a wide range of participants and continuous emphasis on individual *adaptation*.

**Feasibility**. Most patients portrayed YoGI as a feasible intervention due to its suitable length, the mix of different exercises, and its perception as a safe space. Despite different levels of prior experience, all participants deemed the level of difficulty as appropriate, while most interviewed described YoGI as easy. One participant depicts the presented postures as:

“*pleasant, not too difficult. Overarching different age and skill levels. Almost everyone can participate. If one has a physical disability, e.g. being blind, it* [participation] *was difficult; maybe that's a marginal case* [for participation]. *But it's a really large range of people who can participate.” (Informant 5)*

The perception of *feasibility* is also intertwined with the following subtheme *adaptation*, as one participant added:

“*I realised that I probably would not be able to do this* [exercise] *optimally, but I'll do it the way I can, according to my capability and what feels good to my body. And because of that, I did not perceive it* [exercise] *as difficult.” (Informant 25)*

**Adaptation**. While most participants practised the asanas on the mat, four interviewees took part in YoGI while sitting on a chair due to physical limitations. Those participants received tailored cues for their practise (please see YoGI protocol for more details). Everyone was encouraged to follow their own breathing rhythm and individually adapt the postures if needed. Many participants experimented with their body and subsequently adapted exercises, such as a participant who said:

“*Sometimes I had to pause the practise. If one realises it doesn't work at all, or it's too exhausting physically or hurts, it is better to move back into an easier posture for a while*.” *(Informant 8)*

Another participant also expressed appreciation of the focus on adaptation:

“*I liked that one had the freedom to adapt it* [Yoga practise] *to one's own needs. (…). I considered that relaxing knowing it's not about performance but engaging with the postures in a relaxed manner that serves my well-being instead of delivering maximum performance.” (Informant 22)*

#### Challenges

The **challenges** participants experienced throughout YoGI can be divided into difficulties with *engaging in practise* and *physical limitations*.

**Physical Limitations**. A proportion of participants stressed the challenges imposed by physical limitations such as dizziness and overweight, which they noticed while engaging in certain exercises. One participant explained:

“*My arms are so heavy. For a while, I can do the exercise, but then I cannot anymore, because the arms weigh so much. And sometimes, I do not have the strength to hold them up for so long. But I made an effort, and I was very exhausted then.” (Informant 13)*

Many participants also reported awareness of and difficulties due to pain.

**Engaging in Practise**. Participants reported difficulties to start the practise, especially when they did not enter the class in a calm state, and a few mentioned difficulties in understanding the exercises. A crucial factor for interruption of the practise was the presence of others; one participant stated:

“*There were moments in which I was pretty stressed. Not by Yoga but by other patients who always went in and out of the room and rummaged around in their bags or talked. I perceived that as very disruptive.” (Informant 13)*

Moreover, finding one's own breathing rhythm or following the proposed one seemed challenging at times, as one interviewee explained:

“*I don't know, it* [breathing exercise] *doesn't relax me. Mostly it's too slow because I breathe quickly. (laughs) And then I take two breaths while just one* [inhale] *is instructed and then…I don't know.” (Informant 21)*

#### Interconnectedness

The theme **interconnectedness** describes a sense of connection participants felt among each other and with the instructor. The theme arose by combining the subthemes *dialogue with instructor, practising and learning together*, and *feeling connected*.

**Dialogue with instructor**. Participants listened to instructions and observed the instructor entering postures. By instructing the participants to notice bodily sensations and adapt the practise if needed, a dialogue between instructor and participants was created, which one participant described as follows:

“*The therapist raises the question ‘look what it is like for you, whether it feels good to you or whether you need something else' and then one forwards this question to one's own body. And then one answers if one manages to listen to oneself.” (Informant 25)*

This quote demonstrates how this interviewee internalised the instructions and actively engaged with the questions asked by the instructor. Generally, instructions were considered helpful and the empathic attitude in communication was appreciated.

**Practising and Learning Together**. Participants noticed the presence of others and perceived it as helpful to watch and imitate others doing the postures. This subtheme illustrates how a cooperative group atmosphere allowed participants to learn with and from each other. One participant stated:

“*I always looked at you* [instructor] *and then it worked. If one observes someone else, and how this person does the posture, then one can see what you* [instructor] *described, which then makes it relatively easy.” (Informant 8)*

Some participants also compared themselves to other participants and described that practising in a group motivated them.

**Feeling Connected**. Interviewees reported feeling connected to each other as they acknowledged others experiences and appreciated their presence generally. Some communicated that they felt empathy during YoGI. For example, participants said:

“*To some extent, I felt the need that others also keep up with the group, so I observed a little whether they looked happy, too.” (Informant 25)*

“*For me, that's part of Yoga, that it transports something. Like the atmosphere we had today, the participants were all very motivated, wanted to engage in the practise. A good mood, because the people got involved with the class.” (Informant 6)*

#### State of Relaxation

This theme summarises that most participants entered a **state of relaxation** during YoGI. To describe where they felt relaxed, most interviewees referred to the *body:* “*in my body, it wasn't so stiff anymore. It was more relaxed…everywhere, my muscles were eased, more relaxed and thereby I was able to relax and not be so stiff anymore*.” *(Informant 11)*. Others also highlighted the relaxing effect they felt on their *minds*; one participant explained she noticed relaxation by “*[…] not ruminating, having no special thoughts. I would say the relaxation was more in my mind. My mind was relaxed*.” *(Informant 23)*. Other participants expressed their feelings of relaxation in a pictorial manner:

“*Like a fish, I slid into the water. […] Like fish and water belong together, it felt like what exactly is right for one at that moment. And it is something weightless too, that one is unburdened, floating, free of worries. That one is shielded from daily worries and from whatever is burdensome.” (Informant 25)*

Participants experienced some exercises as predominantly relaxing or challenging or both simultaneously. This interplay played a crucial part in entering a state of relaxation as one participant stated that:

“*I can only feel this contrast between tension and relaxation and enjoy the relaxation after I truly was tense.” (Informant 23)*

Another participant emphasises the role of physical engagement and repetition of exercises for relaxation as she stated:

“*I became calmer, so the longer or the more often one does exercises, the more positive the effect will be.” (Informant 18)*

Overall, most participants ascribed a calming effect to the music, and many closed their eyes during parts of the practise.

#### Symptom Representation

The **symptom representation** theme summarises how participants experienced their symptoms as either less straining during YoGI or as impeding the practise.

**Ease of Symptoms**. During YoGI, many participants experienced an increase in well-being, improved pain as well as lifted mood. While many continued to experience auditory hallucinations, interviewees reported less engagement with voices and less strain imposed through these. Generally, during YoGI, participants were distracted from their symptoms and seemed to have fewer thoughts as they focused on the Yoga practise. Moreover, they gained distance to problems and worries, which is pictured by one participant as follows:

“*One is very relaxed and everything else is not so important at this moment: problems, thoughts about what to do tomorrow or in a few hours. Everything stops for a while and it's just about me. […] The strain of problems is gone. One escapes the ‘problem-prison,' and I will just call it like that, leaving stress and pressure behind for a while.” (Informant 2)*

After YoGI, some participants reported improved interactions with others as well as experiencing bliss, a state thought to occur after self-realisation is achieved (58). One participant portrays the following, for instance:

“*When I walk out* [of the session], *I feel my back or shoulder, but in a positive way and think ‘ah what a good day!'. It's as if they* [back and shoulder] *introduced themselves again and became friends, like in an emotionally positive sense. Having everything ‘on board' again, having positive interconnections and that's how I recognise it* [pleasant body feeling]. *I was very grateful for this incredible euphoric feeling of well-being that I was allowed to experience.” (Informant 25)*

**Symptoms Impeding Practise**. Toward the beginning of the session, some participants struggled with staying motivated. During YoGI, feelings of mistrust and hallucinations sometimes prevented full engagement with exercises. However, difficulty concentrating seemed to be the symptom impeding participation in YoGI in most cases:

“*In my head, there was still this mess, back and forth, pushing thoughts around, crisscross, that persisted* [during the exercises]. *That made it kind of difficult to focus on all the exercises.” (Informant 11)*

#### Confidence

**Figure 2:**
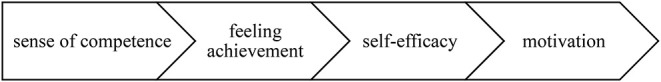
Subthemes of theme “confidence”.

Participation in YoGI facilitated experiencing **confidence** which developed through a *sense of competence* and *achievement*, followed by *self-efficacy* and *motivation*; the four subthemes of this category are displayed in Figure 2.

**Sense of Competence**. Following the flow of asanas required physical perseverance. According to participants, being physically engaged helped to stay focused throughout YoGI. Many interviewees positively acknowledged having sufficient concentration to actively participate whereby they learned new postures. The Yoga class invited participants to try new exercises and challenge themselves which created learning opportunities and consequently, many progressed. The cognitive process of becoming aware of one's learning progress, such as knowing how to adapt a pose suitable for oneself, facilitated experiencing a sense of competence. One participant reviews a moment of progress:

“*The horizontal balance wasn't so easy. That was actually the most difficult thing. It did not work out from the start. I kept trying to hold my leg up and stretch to the front, always a little more and a little more. Until I was somewhat content or rather you* [instructor] *finished the exercise. But I put so much effort into it that I was content then.” (Informant 10)*

**Feeling Achievement**. This sense of competence acquired through the physical practise led many participants to feel proud of their achievement. One participant points out: “*I always like shavasana at the end. It's like you earned it – you do something hard and then you get something sweet as a result*.” *(Informant 1)*. The interviewee indirectly expresses pride at the end of the practise which illustrates the affective component of the confidence theme: to feel achievement. Furthermore, some participants felt flow during YoGI. One participant explains her experience as follows:

“*I did not monitor myself so much anymore. I can only say that there was nothing else except the class. Yes, and for me, that was like a flow. […] I remember when receiving a F-diagnosis that I thought that other people will always be happier than me, that they are healthier and so on. But that was a moment in which I caught myself thinking, ‘right now, at this moment I wouldn't want to change with anyone in this world.”' (Informant 25)*

**Self-efficacy**. By engaging in pranayama and asanas, participants gained insight into the interplay of the breath, body, and mind. For many, Yoga posed an opportunity to learn about themselves, for example, through noticing how one feels today or adjusting postures to a suitable level of difficulty. Furthermore, the physical practise was an empowering experience for some. One participant said:

“*One mobilises powers not knowing that these even played a role. That one consciously concentrates and holds a posture means to challenge oneself and one's body, becoming active to feel better.” (Informant 6)*

Similarly, another participant describes how becoming active through Yoga can lead to feelings of self-efficacy:

“*I like that with Yoga; there are little steps to becoming active again. Not taking 100 stairs at a time but simply paying attention to the breath, noticing how the ribcage expands. Feeling able to do something again, to conquer things.” (Informant 5)*

**Motivation**. Before attending YoGI for the first time, many patients displayed curiosity and motivation to engage in the intervention. Most participants attended YoGI on a regular basis and reportedly left the class feeling motivated to continue their Yoga practise beyond the hospital stay. Additionally, the practise had an activating effect:

“*It* [Yoga] *is relaxation, equanimity for the body…something motivating one in a positive way. One feels better afterwards. Then I also feel motivation to do something else.” (Informant 18)*

“Yoga *motivated me to continue doing sport. It motivated me to do push-ups today and train my abs and maybe I also do squats. It* [Yoga] *motivated me to still do that* [exercises] *today*.” *(Informant 19)*

#### Mindful Stance

**Figure 3:**
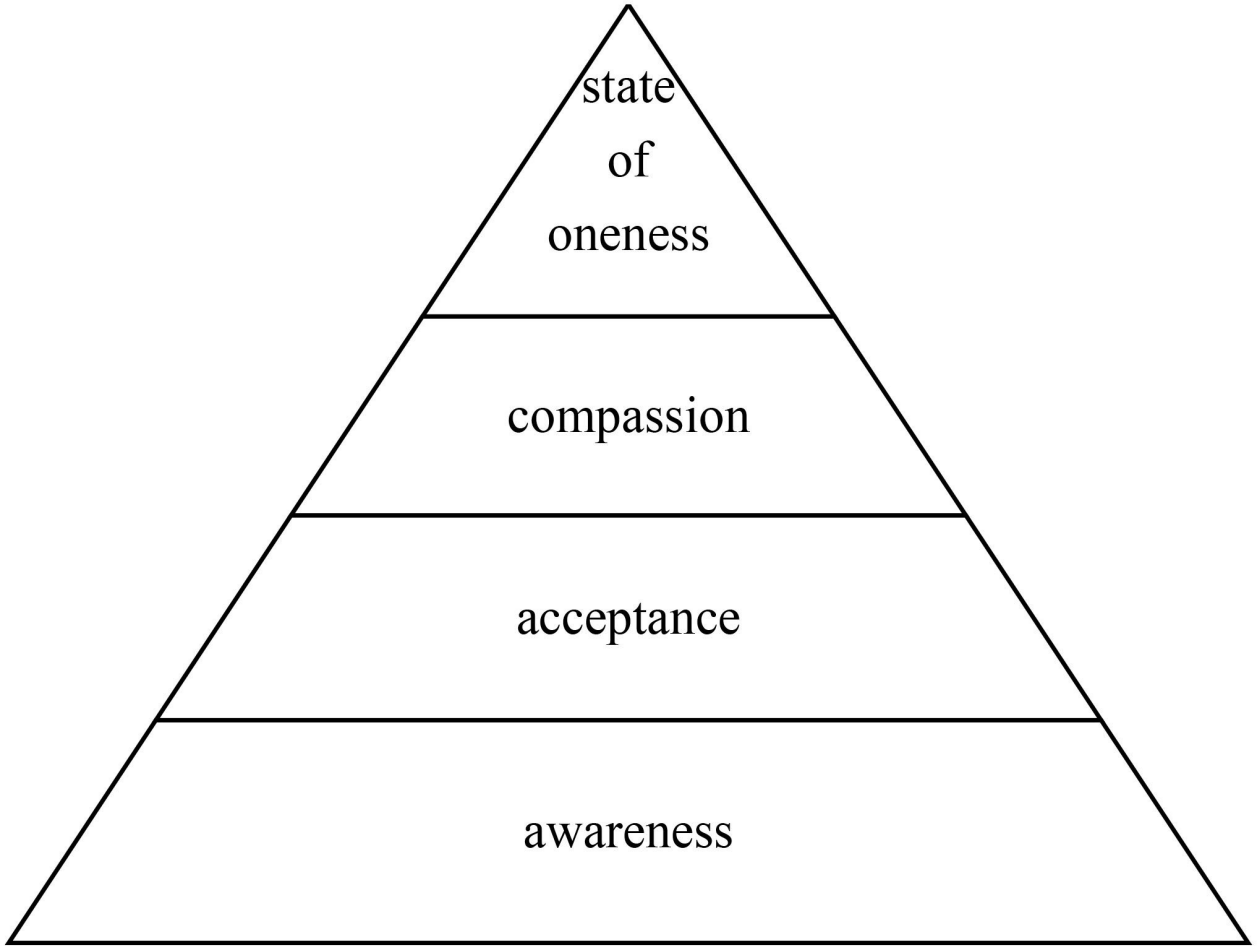
Subthemes of theme “mindful stance”.

The theme **mindful stance** illustrates how participants practised *awareness, acceptance*, and *self-compassion* during YoGI. For some, this resulted in a *state of oneness*. A representation of the subthemes and how these build upon another can be found in Figure 3.

**Awareness**. YoGI guided participants through various exercises, which allowed them to practise awareness of their body, thoughts, and breath. One participant describes heightened awareness of the self through experiencing one's body capabilities:

“*One develops a good feeling for one's body to see what kind of exercises one can do and to feel oneself. I think that's the effect. One concentrates on the body, which poses it can enter and thereby experiences oneself.” (Informant 22)*

**Acceptance**. While many participants were aware of limitations, some pursued this mindfully as they practised acceptance of these and adapted the exercises to their own pace and intensity. One participant expresses how she accepted her own capacities in the face of difficulties:

“*Throughout the whole class, I never felt pressure, neither from you* [instructor] *or in general. […] But there were those brief moments in which I told myself, ‘why don't you manage to balance.' But then I just accepted that is like that or was like that.” (Informant 23)*

**Self-compassion**. Many participants treated themselves with compassion as they listened to bodily sensations instead of forcing themselves into postures. Some utilised Yoga as a form of self-care, such as one participant who illustrated it as:

“*a time in which one takes care of one's well-being and won't get distracted. You* [instructor] *masked the clock so that one does not get side-tracked by the time or other worries and fully concentrates on doing something good for one's body.” (Informant 25)*

**State of Oneness**. The previously described subthemes of **mindful stance** develop in progressive stages, for example, one needs to be aware of pain to be able to accept it and, in turn, accept it to treat oneself in a compassionate manner. For a handful of participants, being mindful led to a *state of oneness*, during which one experiences interconnectedness in the sense of transcending boundaries. One interviewee shares her experience and emphasises the cognitive shift when moving into a state of oneness: “*Then I have completely different thoughts. It's a collective thought and it's not so much about me as an individual anymore*.” *(Informant 19)*. She refers to the group of Yoga practitioners as they posed the immediate environment, however, one could also feel interconnected to nature for instance when experiencing a state of oneness.

## Discussion

The aim of this study was to investigate the experiences of in-patients with SSD who participated in YoGI in a German university hospital. This is the first study to examine the perspective of this patient group in-depth regarding Yoga's underlying processes and its perceived benefits and limitations as a treatment component. Semi-structured interviews were conducted directly after participation in YoGI to ensure detailed and lively descriptions of participants' experiences. In the thematic analysis, seven themes emerged. Overall, YoGI was perceived as feasible and participants appreciated the focus on adaptation, summarised by the theme **inclusivity**. Nevertheless, participants encountered **challenges** during the practise and the theme **symptom representation** captures impeding as well as improved symptoms. The themes **interconnectedness**, **state of relaxation**, **confidence** and **mindful stance** provide insight into the various experiences made by participants during YoGI.

### YoGI: A Feasible and Promising Adjunctive Therapy

When implementing complementary therapy for in-patients with SSD, it is a major benefit that all patients at the ward can participate. Persons with SSD face stigma and discrimination in private and public settings, including psychiatric and health care (59). Participants reported that none of them had been offered YT before, this could reflect the novelty of Yoga as a complementary therapy for patients with SSD, however, it might also point to the delay this patient group experiences in receiving innovative treatment approaches. Thus, an inclusive intervention is of particular importance for this patient group. To ensure **inclusivity** of YoGI, attention needs to be paid to all participants' capabilities and limitations, making adaptability of the Yoga practise a prerequisite. The wide age range of participants (20–64 years) who had different levels of physical and cognitive functioning as well as diverse symptoms representations suggests that YoGI posed a feasible intervention for many in-patients. In addition to *feasibility*, a focus on *adaptation* fosters inclusivity as it eased access to the practise. For instance, participants could follow the Yoga class while sitting on a chair instead of a mat, thereby easing the participation of patients with overweight or old age who initially did not dare to practise Yoga. Furthermore, adaptation offered an accepting attitude inherent to Yoga to deal with arising **challenges** such as inflexibility, pain, or dizziness during YoGI. More importantly, the notion of listening to the body instead of forcing it into poses reduces the risk of injuries and adverse effects. This might increase the safety of the practise while simultaneously promoting agency on the side of the participants.

YoGI's overarching inclusivity and its manageable challenges show that it qualifies as a feasible complementary therapy for patients with SSD. Nevertheless, the practise requires physical exercise and perseverance, which might be of virtue because patients with SSD are significantly less active in comparison to healthy controls (60) as they are faced with complex barriers to uptake physical activities such as impact, especially of negative symptoms of SSD, effects of sedating medication and depression or anxiety (61). Yet, interventions that encourage physical activity are advised for patients with SSD (60, 61), and interviewees stressed how YoGI required physical perseverance, which they treated as a welcome challenge.

In fact, staying physically engaged may put the development of **confidence** in motion: being able to hold postures and having sufficient concentration resulted in a *sense of competence* that was followed by *feelings of achievement*, such as when managing to balance. Learning new asanas and choosing one's level of difficulty set the groundwork for *self-efficacy:* through practising Yoga, participants gained self-knowledge and understood their impact on the interplay of body, mind, and breath. This can pose an empowering experience and may raise self-confidence translating into *motivation* to practise Yoga and engage in other activities (see Figure 1). As lack of motivation constitutes a core symptom of SSD (7), a group therapy with a low barrier to join that leaves participants feeling motivated seems like a promising adjunctive therapy for patients with SSD. In patients with posttraumatic stress disorder YT seemed to set similar processes in motion: themes such as “empowerment” (62) and “feelings of energy and renewal as well” as well as “self-esteem” (63) emerged during the qualitative research processes. This is an example of how Yoga might address the motivational dimension of psychological transdiagnostic processes (64) necessary for fostering human prosperity in line with a process-based care approach that moves beyond a syndromal approach focused on reducing psychopathology (65). In fact, *motivation* and *self-efficacy* could be supportive factors in building personal resources, which alongside NS, predict depression in SSD and, therefore, should be fostered by complementary therapies (66). Also, the clinical relevance of gaining *confidence* should be emphasised since research stresses the importance to strengthen resilience and self-esteem in patients with SSD (67), especially since low self-esteem is closely related to suicide risk in this patient group (68).

On another note, physical perseverance set the stage for an interplay of tension and relaxation, which ultimately led to a **state of relaxation** during YoGI. Except one, all participants reported feeling relaxed at some point during the practise. Achieving a state of relaxation might be especially valuable in the hospital setting (20, 21), where patients are being treated for acute episodes with pronounced symptoms outside of their familiar environment. Previous research has already shown that YT can reduce stress in acutely ill patients at the beginning of their hospital stay (20, 21). Also, a study exploring Yoga experiences of stroke survivors identified the theme “feeling calmer” (69) and “calmness” (70) emerged as a key theme among yoga practitioners, showing that relaxing effects of Yoga span different settings and various participant groups. Further research should follow up these participant reports to investigate the strength and longevity of YoGI's relaxing effects, how these can be utilised and fortified as well as possible side effects.

### How Yoga Affects Symptom Representation

The need for complementary therapies in the treatment of SSD is evident as cognitive dysfunctions and NS are not effectively targeted by current treatment while being experienced as enduring and persistent symptoms by people with SSD (34). Severe NS can impede participation, yet YoGI shows potential to adjust to and affect some of the five constructs of NS; blunted affect, diminished speech, anhedonia, social withdrawal, and lack of motivation (7). For example, diminished speech did not seem to pose a barrier to practising Yoga. Nonetheless, practitioners were verbally engaged as they were asked to briefly share their intention at the beginning of the session. This might have created an explicit dialogue in addition to the connexion evolving through the non-verbal shared consciousness of practising Yoga together, thereby offering an opportunity for social relations. While many patients with SSD experience a lack of motivation, most participants at the ward managed to attend YoGI regularly. Furthermore, after YoGI, some left feeling motivated to engage in an additional individual Yoga practise or engage in other tasks reflecting the effect the intervention had on anhedonia. Interviewees mentioned that they experienced elevated mood and positive emotions, such as joy, during as well as after the Yoga class. The mechanisms underlying this effect of YoGI are not yet established, thus study designs involving a control group that assess a variety of external factors need to follow up the reported experiences to be able to establish causal relationships. However, similar processes have been observed with mindfulness practises showing an increase in positive emotions and anticipatory pleasure (71). Since a lack of anticipatory pleasure has been linked to NS (72, 73), interventions facilitating these processes might indirectly target clinical manifestations of SSD. Moreover, YoGI might have a preventative effect on negative mood spirals, similar to a reduction in negative thinking that has also been observed as a mechanism of mindfulness (74). During YoGI, the asana practise requires one to notice one's body in a mindful manner, thereby providing an activity that demands continuous attention to inner sensations, preventing participants from engaging in rumination that could maintain and intensify low mood (75). Yet, randomised controlled trials of YoGI are needed to investigate the sustainability of the proposed effect. Some participants continued to experience auditory and visual hallucinations during YoGI. However, voices were perceived to be less frequent, quieter, and participants reported less engagement and less strain imposed through these. This observation is in line with the rationale for applying mindfulness to distressing psychotic sensations (76). According to the model, distress may arise as one is not able to notice and accept psychotic (and any other possibly unpleasant) sensations as temporary experiences, instead, a *mindful response* involving clear awareness could pose a more adaptive reaction. Most importantly, there was no increase in hallucinations or other PS consistent with previous research on Yoga and mindfulness-based interventions, showing that these are safe interventions for patients with psychosis (77–80). Additionally, patients seemed to feel safe as only very few reported incidents of mistrust of others, such as feeling nervous or watched, or not being able to close their eyes during Yoga. This outcome is particularly noteworthy since mistrust constitutes one of the core symptoms of SSD. As a prerequisite, instructors need to ensure that the group and the room setting is experienced as a safe space allowing participants to fully engage in YoGI. In this environment, the majority experienced a feeling of **interconnectedness**. Also, Visceglia (81) describes examples of how people with schizophrenia felt connected to others during the Yoga practise and underlines how interconnectedness can enhance well-being (82). In qualitative YT studies with other patient groups themes that can broadly be categorised under “connection” regularly emerge: “relatedness” was found in patients with posttraumatic stress disorder practising Yoga (62). In addition, “belonging” and “sustaining community connection” was reported by YT participants who had a traumatic brain injury (83) and “becoming connected” arose as a theme among stroke survivors joining a Yoga programme (69). Although while practising Yoga participants usually do not directly interact, interviewees reported that they became aware of others presence through observing each other entering poses. Despite the absence of direct verbal exchange, practising and learning together created a supportive group atmosphere. This non-verbal shared consciousness can facilitate equal or even greater benefit than verbally shared consciousness (84).

### Yoga—An Embodied Form of Mindfulness?

The theme **mindful stance** (see Figure 3) shows the progressive development of mindfulness during YoGI: Most participants were able to become aware of bodily sensations, many adopted an accepting attitude toward these, and for some, this resulted in treating oneself more compassionately. Similar experiences have been reported from patients with pain who participated in YT and describe “renewed awareness of the body” (85, 86) and “increased acceptance of pain and disability” (86) as well as a “transformed relationship with the body in pain” (85). These examples illustrate Yoga's inherent focus on enhancing body awareness which constitutes Yoga as a practise that integrates an accessible form of mindfulness. Through mindful instructions, participants are guided to (re-)direct their attention to notice any bodily sensations, which corresponds to continuous access to bottom-up sensations necessary for “*embodied mindfulness*” (47). In the conceptualisation of *embodied mindfulness*, present moment awareness, a core element of mindfulness, emerges through the interaction of top-down and bottom-up processes that underlie the unfolding of any conscious experience in the here and now (47). In YoGI, participants engage in this interaction as they assess to what extent entering a specific asana might be too intense or even hurt *(bottom-up process)* and based on this sensation, they might adapt the pose to their capabilities and liking *(top-down process)*. By continuously integrating bottom-up and top-down processes, some participants experienced a *state of oneness*, also known as “*the sense of calmness, equanimity, and peace associated with meditative practices*” (87).

**Figure 4:**
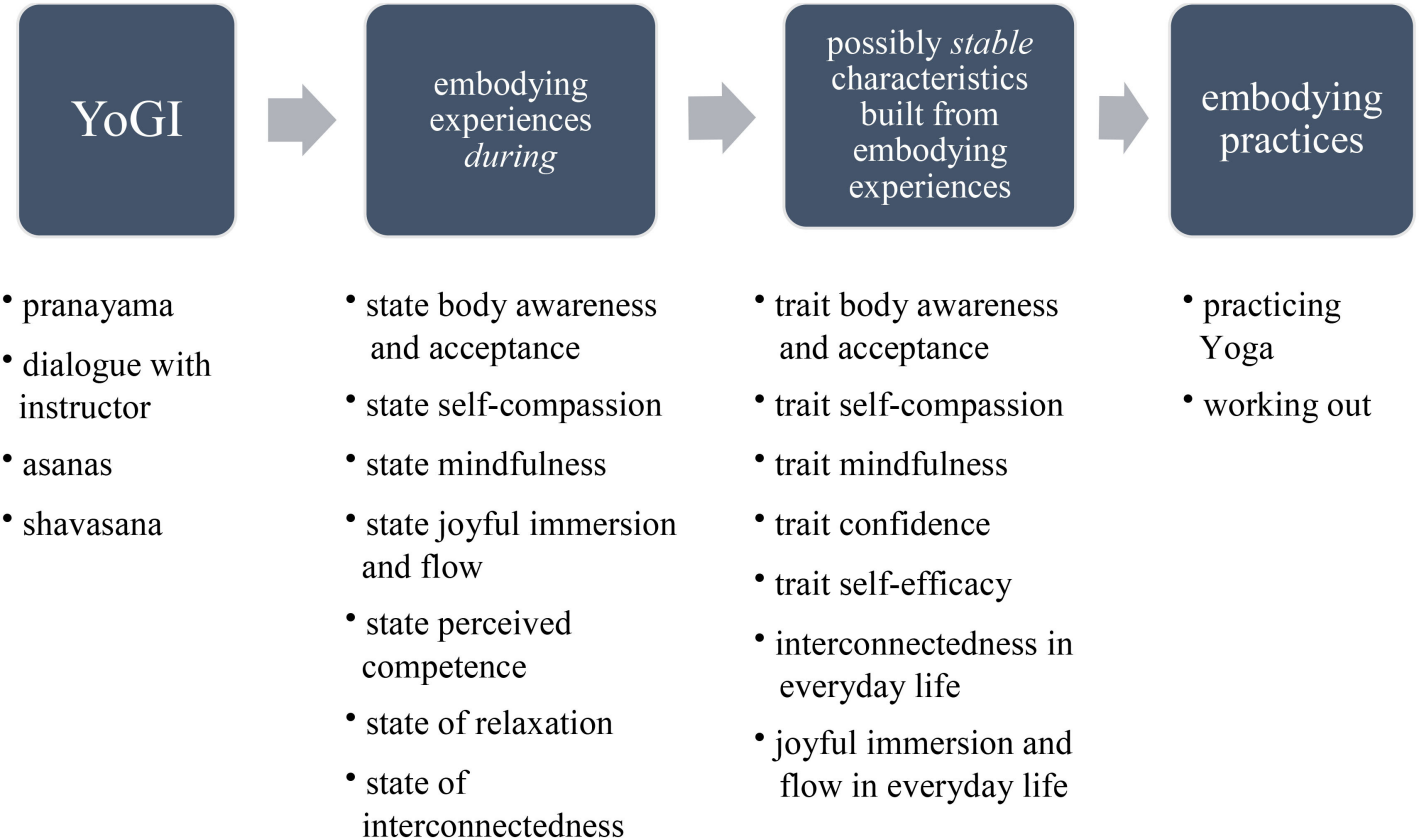
Model of how Yoga may promote positive embodiment by Cox and Tylka (89) fit to YoGI and its proposed underlying mechanisms.

The **mindful stance** adopted during YoGI, involving *awareness, acceptance*, and *self-compassion*, might have the potential to extend beyond the Yoga class, as participants of a long-term Yoga intervention showed a significant increase in trait mindfulness (88). Further research should investigate if and how mindfulness practise acquired through YoGI can be utilised to enhance well-being and advance current treatment approaches. A model by Cox and Tylka (89) proposes how Yoga may promote positive embodiment through mindfulness, possibly lasting beyond the Yoga class: First, Yoga cultivates embodying experiences that can be understood as being in a state of mindfulness. Second, by building on these embodying experiences, people might develop trait mindfulness over time. Third, being more mindful might promote engaging in embodying practises such as mindful self-care. Participants of YoGI expressed how their participation and the dialogue with the instructor activated them to engage in self-care beyond the Yoga class (see Figure 4). Moreover, a large proportion of participants regularly joined YoGI and showed interest in continuing their Yoga practise after discharge from the hospital. This reflects a wish to continue mindful practises and shows the potential of YoGI to become a complementary therapy sought out by patients, possibly bridging the gap between in- and outpatient treatment.

### Limitations and Strengths

A limitation of this study is the use of a convenience sample as this enables selection bias. It might be that those who chose to participate in YoGI, as well as the study, had a more favourable attitude toward Yoga in the first place. Their voluntarily participation indicates motivation to practise Yoga even before the intervention and assessment, thus, the described therapeutic effects cannot be ascribed to participation in YoGI. The interviewer was involved in co-instructing YoGI, which makes the responses susceptible to social desirability bias. Hence, results need to be regarded with caution and should be followed up in further research systematically separating tasks of instructors and interviewers to avoid social desirability bias on the side of participants. Furthermore, one of the researchers was involved in the interviews and the thematic coding process; thus, it cannot be ruled out that the researcher's preconceptions of YoGI might have influenced the participants' answers and interpretation of data. The interview was conducted directly after YoGI and usually, participants were interviewed after visiting their first session and latest after no more than three sessions. It might be that different themes relating to practise and long-term effects would have formed if participants were interviewed after visiting several sessions of YoGI. However, neither the exact number of sessions nor the total number of YoGI sessions attended was recorded. In addition, dosage details of medication, education, and socioeconomic status were not assessed. Yet, these factors might change how participation in a Yoga-based group intervention is experienced and hence, these should be systematically assessed in future studies.

Moreover, generalisations of the described findings to other settings and patients cannot be made. The setting of YoGI poses an important factor as the ward itself provides a safe space and most participants knew each other beforehand. Hence, different experiences might arise if YoGI would be offered in, for example, an outpatient setting. Additionally, patients with severe positive symptoms of psychosis were excluded from participation (any item from the P-scale of the PANSS rated equal to six or higher). Thus, preventing any generalisations of the reported experiences to those experiencing severe psychotic symptoms or not receiving treatment in an in-patient setting. An area for improvement poses the delivery of YoGI as the psychologists who instructed the intervention did not complete a certified Yoga teacher training yet. However, the YoGI protocol was developed in collaboration with a team of psychologists and trained Yoga teachers who guided psychologists in instructing YoGI until they resumed instruction of the intervention. These limitations can, however, be seen alongside several strengths. The semi-structured interview guide was pilot tested during a period of five interviews; hence questions were optimised regarding understandability and appropriateness. In the data collection and analysis process, data saturation has likely been achieved after conducting 25 interviews (53). During data analysis, two researchers independently analysed the data to prevent the impact of subjective bias and strengthen the reliability of data coding and interpretation.

The present study is the first to offer in-depth insights into the experience of patients with SSD practising Yoga in a German hospital setting. The detailed feedback interviewees provided on sessions allowed continuous adaptation and refinement of the intervention that provides a first basis for developing a YoGI manual in the future. Furthermore, given participants' overall positive experience of the intervention, a randomised controlled trial is planned to systematically investigate the size and longevity of YoGI's various effects.

## Conclusion

The present study is the first to describe in-depth experiences of patients with SSD who participated in YoGI in a German university hospital setting. The qualitative approach allowed participants to report their experiences in detail, thereby exploring underlying mechanisms of YoGI as well as its benefits and limitations in the treatment of SSD. Based on the outcomes, one can conclude that YoGI qualifies as a feasible and promising complementary therapy for this patient group. However, physical limitations need to be taken into consideration, and individual adaptation should be encouraged to ensure inclusivity of the Yoga practise. In addition to the positive effect on the symptomatology of in-patients with SSD, YoGI increased many psychological resources such as confidence and mindfulness. Moreover, among a patient group that experiences high levels of stress and has social interactions and relationships possibly impeded by mistrust, Yoga seemed to have particularly positive effects: Most participants experienced a state of relaxation and interconnectedness while practising Yoga. To what extent the various effects of YoGI can be sustained and how participants might transfer the mindful approach to the Yoga practise to their lives off the mat should be examined in future research.

## Data Availability Statement

The raw data supporting the conclusions of this article will be made available by the authors, without undue reservation.

## Ethics Statement

The studies involving human participants were reviewed and approved by the ethics committee of the Charité Universitätsmedizin Berlin. The patients/participants provided their written informed consent to participate in this study.

## Author Contributions

TS designed and executed the study, conducted the data analyses, and wrote the paper. EH collaborated with the design and editing of the final manuscript. IH and NB collaborated with the design and execution of the study and editing of the manuscript. LF and GP assisted with the study design and edited the manuscript. FM assisted with conducting the data analyses. MZ assisted with the editing of the final manuscript. TT collaborated with editing the final manuscript. KB designed the study and edited the manuscript. All authors contributed to the article and approved the submitted version.

## Conflict of Interest

The authors declare that the research was conducted in the absence of any commercial or financial relationships that could be construed as a potential conflict of interest.

## Publisher's Note

All claims expressed in this article are solely those of the authors and do not necessarily represent those of their affiliated organizations, or those of the publisher, the editors and the reviewers. Any product that may be evaluated in this article, or claim that may be made by its manufacturer, is not guaranteed or endorsed by the publisher.

## Appendix

**TABLE A1:** Guideline for the semi-structured interview.

1. Could you describe what first comes to your mind when thinking of the Yoga-based group intervention?
2. Do you have any previous experience with Yoga, or does it remind you of other exercises?
3. How would you characterise the exercises?
4. What did you feel during the exercises?
5. How and what did you perceive during the exercises?
6. Did you notice any challenges or difficulties during the exercises? If so, which ones?
7. Which exercise did you dislike and why?
8. What did you manage well and what did you like?
9. Which exercise did you like best and why?
10. Did the exercise affect you anyhow? If so, how?
11. What can you take away for yourself from this exercise?
12. What did you miss? What could have been done better or differently?
13. What do you think of the room, structure and group size of the Yoga-based group intervention?
14. What did you perceive regarding your symptoms during and after the exercises? Time for remarks and comments
