# Supplementary material for: Yoga-Based Group Intervention for In-patients With Schizophrenia Spectrum Disorders—A Qualitative Approach
Source: Front Psychiatry. 2021 Aug 13;12:715670. doi: 10.3389/fpsyt.2021.715670 (PMC8414901; doi:10.3389/fpsyt.2021.715670)
Supplement: Supplementary file 1 [file Data_Sheet_1.docx]

# Exemplary protocol of the Yoga-based Group Intervention (YoGI)

1. **Introduction** (~2 minutes)

- Instruct participants to listen to themselves & their own body foremost!
  - *‘You can follow instructions of poses and any variations offered. But you know yourself and your body’s abilities best, thus, always feel free to adapt poses so they feel good to you.’*
- Remark to decrease any worries that participants may have: Many think that Yoga is about fancy poses and worry about being flexible or strong enough for the practice. However, that does not reflect the essence of Yoga and none of that is requested here. Instead, while practising Yoga try to replace the thought of *‘having to perform good’* with *‘doing something good for myself’.*
- Explain that adaptions can be made anytime and offer options to rest or slow down: *‘When you feel overwhelmed, need to catch your breath or want to take it more slow you can always come back to child’s pose (balasana) or just lie on your belly or back and pay attention to your breath.’*

1. **Breathing Exercises *(pranayama)*** (~10 minutes)

- Guide participants into a comfortable sitting position: e.g. cross-legged seat *(sukhasana)* or staff pose *(dandasana)*.
- Start with a brief breathing exercise: *‘While breathing in raise shoulders to ears, breathe out loudly through your mouth and let shoulders sink.’* (repeat 5x)
- Guide participants attention to observe their breath:
  - *‘Watch how your breath comes and goes in its own natural rhythm.’*
  - *‘There is no need to slow your breath down or deepen it right now, just observe the flow of your breath as it is.’*
  - Give focus points such as feeling into the tip of one’s nose, focussing on how the air moves in and out of the nostrils with every breath and observing the sensation of feeling the temperature of the breath (a little warmer with the exhalation and taking in cooler air with the inhalation).
- Guide participants attention to notice their thoughts, feelings, and/or body. Example for the body:
  - Start by instructing an upright seating position: *‘Hold your pelvic upright and let it become heavy. From here relax your legs, knee and feet. Do not hold any tension in your lower body and let it sit firmly on the floor, resting comfortably. At the same time your spine reaches upward, create length from your lower back over your neck up to the top of your head. Feel how you are sitting upright and notice the length in your upper body. Breath and create space to become a little taller with every inhale and ground yourself firmly to the floor with every exhale.’*
  - Let participants notice which body parts they noticed. Is there tension somewhere Does a specific body part feel light or maybe hurt? What part do they want to give special attention to in today’s Yoga session? Ask participants to set an intention for class, such as breathing consciously, strengthening the back, resting, being open for arising sensations etc.
  - Brief round for participants to share their intentions. This round can also be used to inform the instructors about the mood of participants as well as special requests and physical limitations of participants.

1. **Physical practice *(asanas)*** (~30 minutes)

- seated position:
  - slowly rolling neck down to chest and from side to side
  - activating spine: *Sufi* circles
  - seated spinal twist *(parivrtta sukhasana)*
  - child’s pose *(balasana)*
  - cat/ cow pose (marjaryasana / bitilasana)
  - all-fours position with active options:
    - stretch out left/right arm and right/left leg (practice balance)
    - bring left/right arm and right/left knee together underneath belly (core activation)
  - child’s pose *(balasana)*

*remark:* For those taking part while sitting on a chair the seated spinal twist and cat/ cow pose can be practiced similarly while seated on a chair, even including the active options. For child’s pose participants can either rest chin on chest and close eyes or find another seated posture in which they can relax.

# standing position:

- - Take time to explain a stable, upright standing position from toe to head *(tadasana).*
  - chair pose (*utkatasana)* in interchange with *tadasana*
  - warrior I *(virabhadrasana I)* in adapted versions
  - warrior II *(virabhadrasana II)* in adapted versions, if time include backbends and forward folds
  - tree pose *(vrikshasana)*, integrate brief balancing exercises before to demonstrate weight-shifting

*remark:* For those taking part while sitting the instructions for *tadasana* can be adopted for an upright seated position. Instead of holding *utkatasana* participants can feel and tense the muscles needed as if they would want to get up from the chair. For warrior poses participants can take a stable position in their feet and legs, stretch their arms to the side and feel into the power and confidence activated by warrior poses. For tree pose participants can balance with one foot while sitting or, if possible, stand up and balance while holding onto the chair.

- lying position:
- lie down on belly:
  - cobra *(bhujaṅgāsana)*
  - grasshopper *(śalabhāsana)*
  - Move hips from side to side to loosen lower back.
- turn around, now lying down on back:
  - shoulder bridge *(setu bandha sarvāṅgāsana)*
  - Draw knees to upper body and move with hips and back from side to side, maybe grab knees with your hand and draw circles to massage your lower back.
  - twist *(jathara parivartanasana)*

# **Relaxation *(shavasana)*** (~8 minutes)

- Turn off or dim lights.
- Invite participants to close eyes for relaxation (only if that feels comfortable for them, otherwise they can also leave eyes open and let their gaze soften).
- Invite participants to see if with every exhale they can let go of more tension and let their bodyweight keep sinking down to the floor.
- Instruct a body scan in which participants are being talked through feeling into specific body parts separately, starting from head to toe or the other way around. Participants can be instructed to relax part by part or simply feel into certain areas and notice what sensations come up. During the body scan (as well as the whole class) total silences should not span much more than a couple seconds.
- Slowly come back up again (eyes still closed if possible).

1. **End** (~2 minutes)

- Ask participants to put hands together in *namasté*: *‘Thank yourself for taking the time to practice, thereby nurturing your mind, body & soul! Thank you for coming today and sharing the space to practice with others and maybe see how you can be attentive to yourself and your needs outside of the Yoga class, too.’* Bow down, invite participants to slowly open eyes and announce end of Yoga session.
- Participants can take some time on the mat to slowly arrive back in the room and do any movements or stretches that intuitively feel good to them.
- Offer participants time to ask questions they may have and space to voice any remarks.
